# Polysomnographic Analysis in Snoring Patients

**DOI:** 10.7759/cureus.84018

**Published:** 2025-05-13

**Authors:** Yan Li

**Affiliations:** 1 Otorhinolaryngology, Shanghai Yida Hospital, Shanghai University, Shanghai, China; 2 Otorhinolaryngology, Wuxi Huawei Hospital, Wuxi, CHN

**Keywords:** body mass index, hypoxia, obstructive, psg, severity of illness index, sleep apnea, snoring

## Abstract

Objective: This study aimed to investigate differences and correlations in polysomnography (PSG) parameters among snoring patients in the otolaryngology department by gender, age group, and apnea-hypopnea index (AHI) severity level.

Methods: Differences in parameters (AHI, oxygen desaturation index (ODI), snoring index (SI), body mass index (BMI), and age (AGE)) were compared across genders, age groups, and AHI severity levels. Relationships among these parameters were analyzed using bar charts, box plots, and correlation matrices to characterize snoring patients.

Results: Under the same BMI, male patients exhibited significantly higher values for AHI, ODI, and SI compared to female patients (p < 0.01). Both AHI and ODI increased with age, showing significantly higher values in elderly patients (≥60 years) compared to younger patients (<40 years) (p < 0.01). No significant differences in SI were observed across different AHI severity groups. At moderate AHI levels, ODI demonstrated the strongest positive correlation with BMI (r = 0.58) and the strongest negative correlation with SI (r = -0.61). AHI was positively correlated with both ODI and BMI, with the strongest correlations observed at severe AHI levels (r = 0.84, r = 0.49, respectively).

Conclusion: The systematic analysis of these parameters provides a more comprehensive and scientifically grounded reference for early diagnosis, personalized treatment planning, and dynamic assessment of snoring conditions.

## Introduction

Obstructive sleep apnea-hypopnea syndrome (OSAHS) is a prevalent sleep-related breathing disorder, characterized by recurrent episodes of apnea and hypopnea during sleep due to upper airway obstruction [1]. It affects a significant portion of the global population, with prevalence rates varying between 2% and 10% worldwide. Notably, the incidence of OSAHS increases with age, with a higher prevalence observed in individuals over 65 years old, particularly in men, where the prevalence can reach up to 24% [2]. OSAHS is strongly associated with numerous health risks, including but not limited to respiratory difficulties, impaired sleep quality, cardiovascular diseases, metabolic disorders, respiratory infections, gastroesophageal reflux disease, mood disturbances, sexual dysfunction, and an elevated risk of accidents [3-8]. Consequently, the early detection and effective management of OSAHS are essential for enhancing patients' quality of life and mitigating potential complications [4,6,9].

Polysomnography (PSG) is universally recognized as the gold standard for diagnosing OSAHS [10]. In this study, we employ a 22-channel PSG device developed in Beijing, China. This advanced device enables real-time tracking and recording of multiple physiological parameters, thereby providing robust data support for evaluating patients' sleep quality and associated pathological characteristics.

This study aims to establish a more comprehensive and scientifically grounded reference framework for the early diagnosis, personalized treatment planning, and dynamic monitoring of OSAHS by systematically analyzing the differences and correlations among key parameters, including the apnea-hypopnea index (AHI), oxygen desaturation index (ODI), snoring index (SI), body mass index (BMI), and age (AGE), across various demographic groups defined by gender, age, and AHI severity levels [11-13].

## Materials and methods

Ninety patients diagnosed with snoring by PSG in the Otolaryngology Department of Shanghai Yida Hospital, Shanghai, China, between January 2023 and January 2024, were recruited as subjects, and their clinical data and PSG parameters were collected. The inclusion criteria included patients aged six to 80 years with suspected OSAHS, Epworth Sleepiness Scale (ESS) score≥9 [14], no conditions significantly impacting sleep or respiratory function, and who provided informed consent for PSG evaluation and follow-up. The exclusion criteria are patients with central sleep apnea, non-snoring-related sleep disorders, anatomical abnormalities affecting assessment accuracy [15], medication use impacting sleep or respiratory function, or inability to complete PSG monitoring. Using IBM SPSS Statistics for Windows, Version 27.0 (released 2020, IBM Corp., Armonk, NY) to test the power value (power > 0.8), the sample size has a high probability of detecting the real differences between parameters. This study was approved by the Ethics Committee of Shanghai Yida Hospital (approval number: 2023-0101). All patients were informed of the study and provided their written informed consent.

Methods

Patients were grouped by age (0-20, 20-40, 40-60, 60-80 years), gender (male, female), and AHI severity (normal: zero to five times/hour, mild: five to 15 times/hour, moderate: 15-30 times/hour, severe: >30 times/hour [16]).

Statistical software (Python and Jupyter Notebook in Anaconda3, Anaconda, Inc., USA) was used to analyze parameters (AHI, ODI, SI, BMI, and age) across groups. Methods included bar charts for mean comparisons, box plots for distributions, and heatmaps for correlations. The Pearson correlation coefficient assessed linear relationships, with values indicating weak (0.1-0.3), moderate (0.3-0.5), strong (0.5-0.7), and very strong (0.7-1) correlations [17].

## Results

Gender differences in PSG parameters

**Figure 1:**
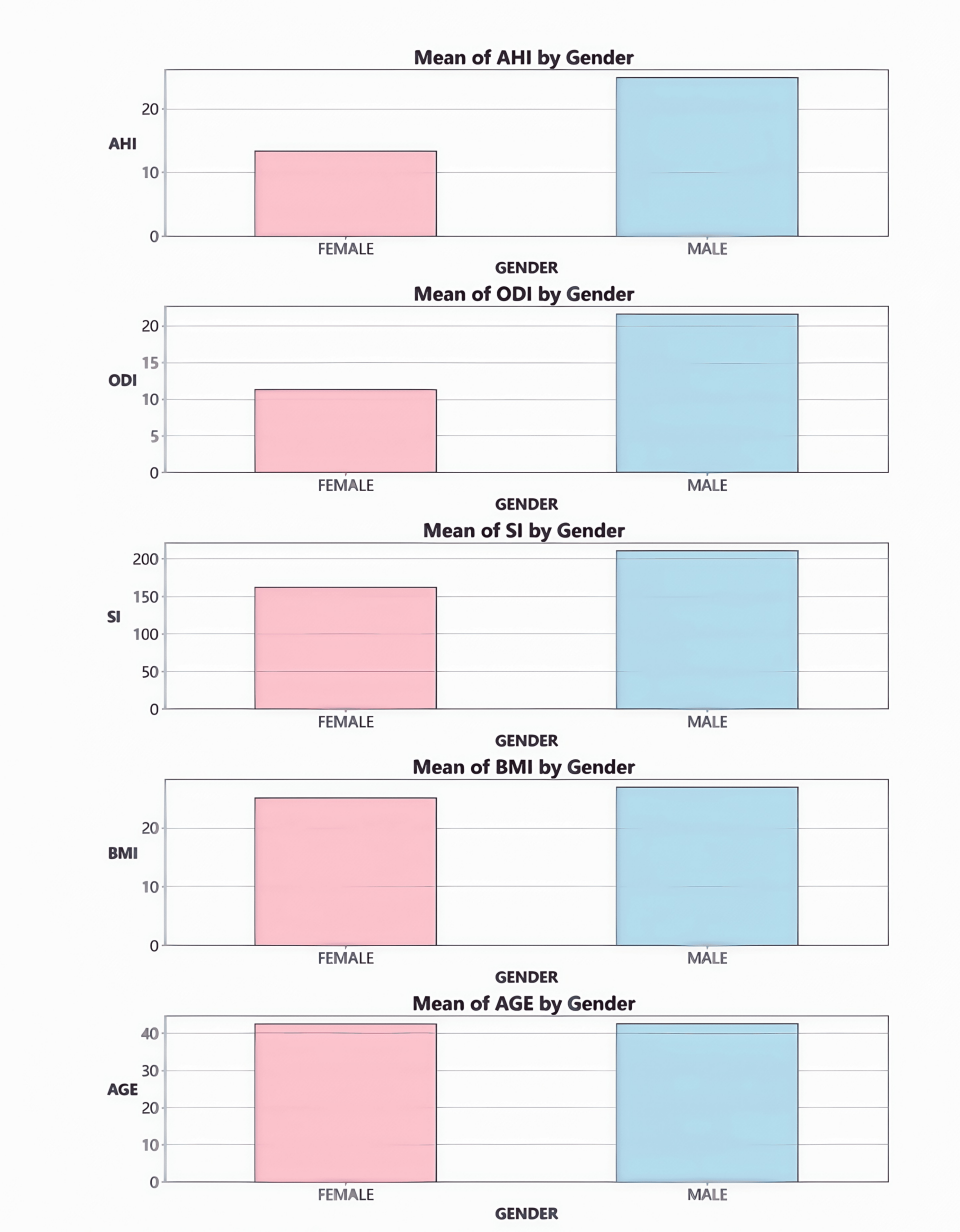
Bar chart of average parameter values by gender Created with statistical software Python and Jupyter Notebook in Anaconda3 (Anaconda, Inc., USA)

The bar chart (Figure 1) vividly illustrates the disparities in PSG parameters between genders. Notably, male participants exhibited significantly higher values in AHI, ODI, and SI compared to female participants (p < 0.01). It is important to highlight that, within this study, no significant gender differences were observed in the distribution of BMI and age (p > 0.05).

PSG parameter differences by age group 

**Figure 2:**
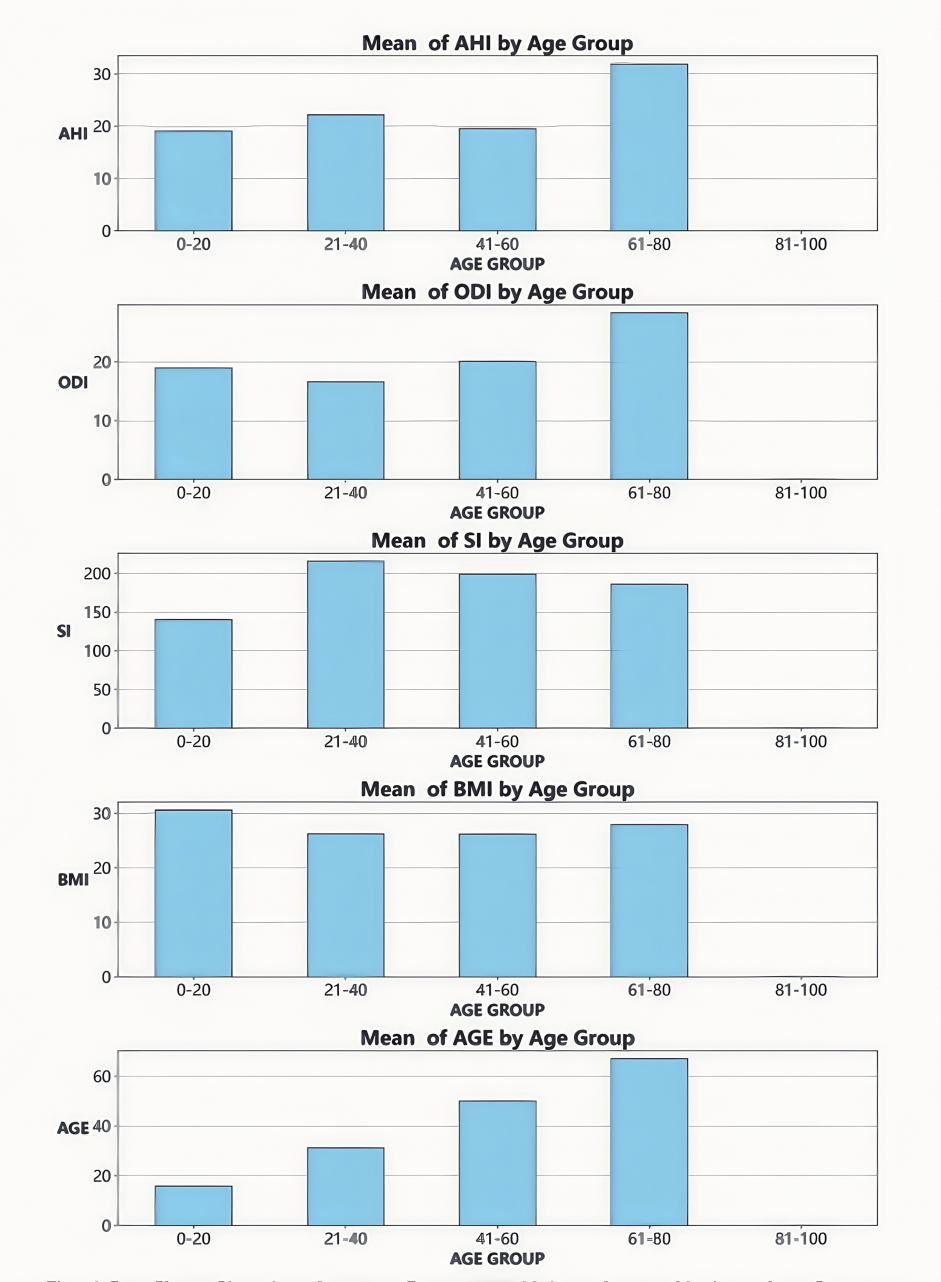
Bar chart showing average parameter values across various age groups Created with statistical software Python and Jupyter Notebook in Anaconda3 (Anaconda, Inc., USA)

The bar chart（Figure 2) illustrates the variations in PSG parameters across different age groups. AHI and ODI exhibit an increasing trend with age, reaching their highest levels in the 60-80 age group and the lowest in the 0-20 age group. SI is relatively elevated in the 21-40 age group; however, no significant differences are observed among age groups. BMI is slightly higher in the 0-20 age group, yet no statistically significant differences exist across age groups. AGE differences are defined by the grouping criteria and serve to validate the accuracy of the classification.

In summary, age demonstrates a positive correlation with AHI and ODI, with significantly higher values observed in the elderly group (≥60 years) compared to the young group (<40 years) (p < 0.05).

Differences in PSG parameters across groups by AHI severity

**Figure 3:**
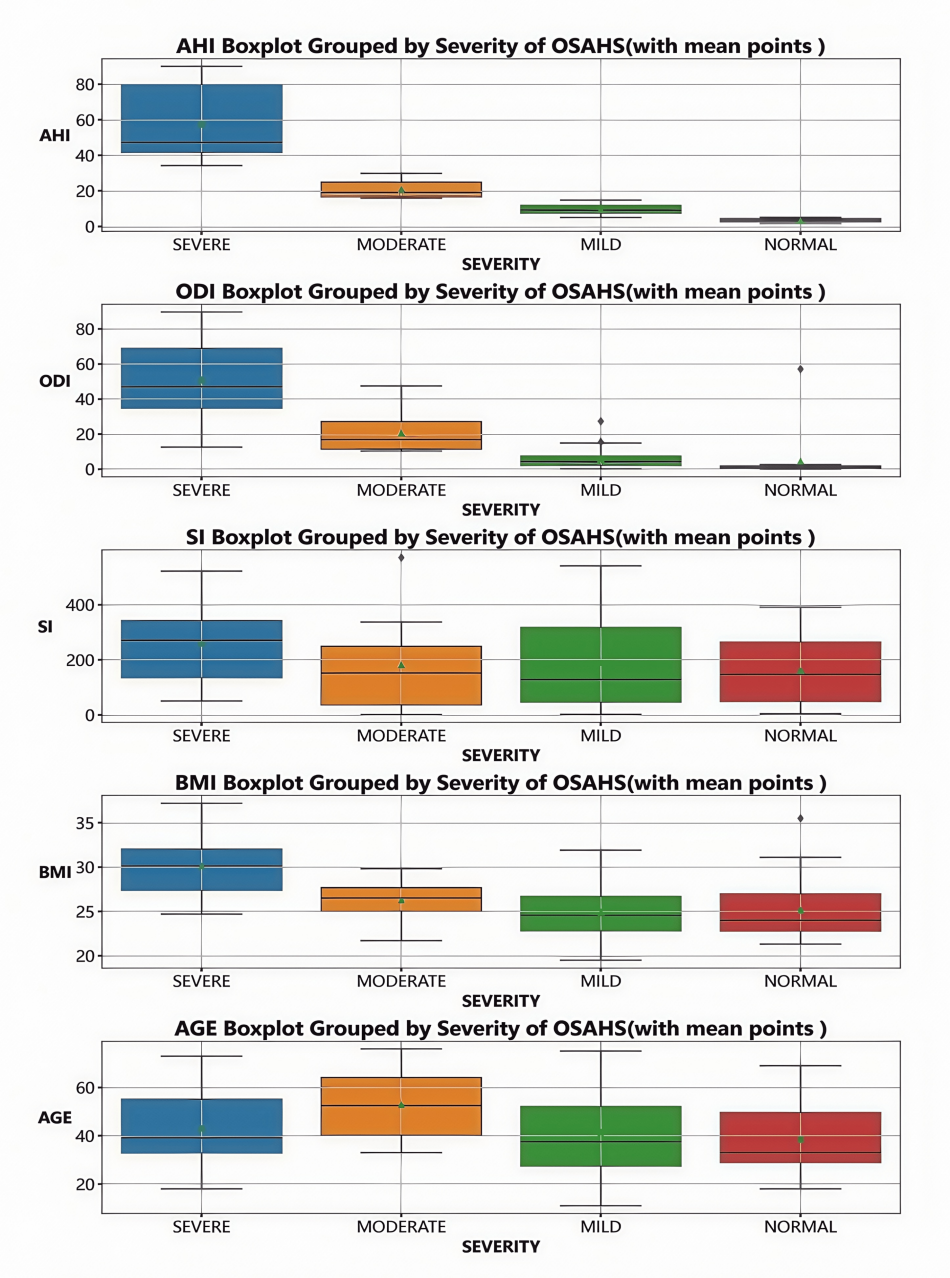
Box plot illustrating parameters across various OSAHS severity levels OSAHS: obstructive sleep apnea-hypopnea syndrome Created with statistical software Python and Jupyter Notebook in Anaconda3 (Anaconda, Inc., USA)

The differences in PSG parameters among different AHI severity groups were presented by box plots (Figure 3). ODI and BMI gradually increased with the severity of AHI and reached the highest values in severe AHI. There was no significant difference in SI among the groups. Although there was no obvious pattern in the age distribution, moderate AHI was more common in the elderly.

Correlation matrix of parameters across AHI levels

**Figure 4:**
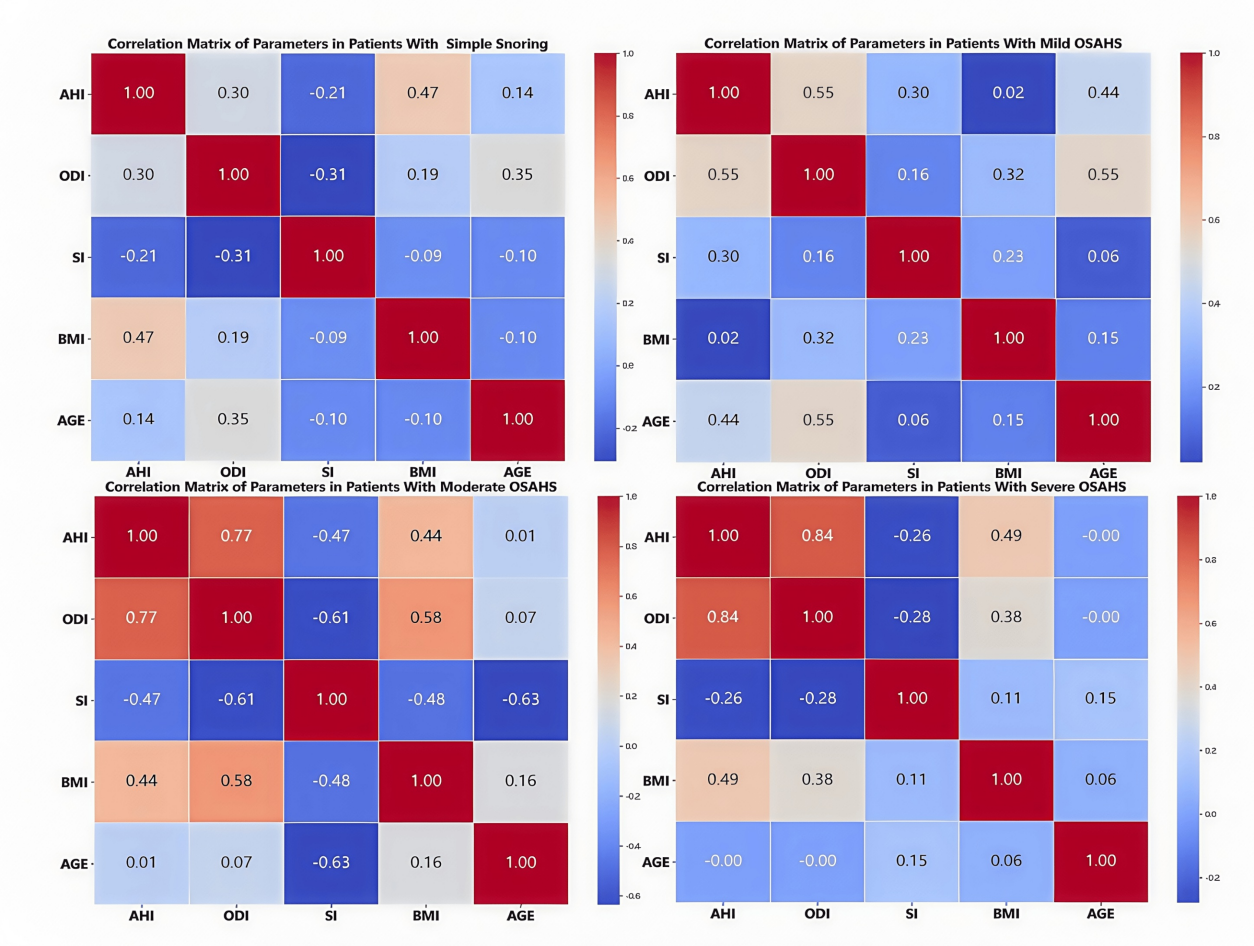
Linear correlation among parameters at various OSAHS severity levels OSAHS: obstructive sleep apnea-hypopnea syndrome Created with statistical software Python and Jupyter Notebook in Anaconda3 (Anaconda, Inc., USA)

Based on the AHI grouping and matrix chart analysis, the correlations among parameters are summarized as follows (Figure 4): 

AHI and Other Parameters

AHI is positively correlated with ODI (strongest at severe levels, r = 0.84). AHI is positively correlated with BMI (strongest at severe levels, r = 0.49). AHI correlates most strongly with AGE at mild levels (r = 0.44). AHI correlates positively with SI at mild levels (r = 0.3) and negatively at moderate levels (r = -0.47). 

ODI and Other Parameters

ODI is positively correlated with AHI. At mild AHI, ODI correlates most strongly with AGE (r = 0.44). At moderate AHI, ODI correlates most strongly with BMI (r = 0.58) and negatively with SI (r = -0.61). 

SI and Other Parameters

SI correlates negatively with AGE (r = -0.63), positively with ODI (r = 0.61), negatively with BMI (r = -0.48), and negatively with AHI (r = -0.47). 

Age and Other Parameters

At mild AHI, AGE correlates most strongly with ODI (r = 0.44). At moderate AHI, AGE correlates most strongly negatively with SI (r = -0.63). 

BMI and Other Parameters

For the BMI-AHI correlation, mild was r = 0.47, moderate was r = 0.44, and severe was r = 0.49. For the BMI-ODI correlation, moderate was r = 0.58 and severe was r = 0.38. For the BMI-SI correlation, the strongest negative at moderate levels was r = -0.48. 

In summary, AHI correlates positively with ODI and BMI, while its relationships with AGE and SI are complex. Correlations vary by AHI severity, with significant findings under specific conditions.

## Discussion

Gender factor

Gender differences are particularly pronounced in patients with sleep apnea. Specifically, when BMI is matched, male patients exhibit significantly higher mean values of AHI, ODI, and SI compared to female patients (p < 0.01). This gender disparity may result from multiple factors. From an anatomical and physiological perspective, men typically have a narrower upper airway structure than women, which increases the likelihood of airway obstruction. In addition, lifestyle differences may also play a critical role in the onset and progression of sleep apnea. For instance, men are more likely to smoke and drink alcohol, habits that can exacerbate airway inflammation or impair respiratory regulation. Hormonal variations are another potential influencing factor; studies indicate that estrogen may protect against airway collapse, thereby reducing the risk of sleep apnea in women. Thus, a deeper understanding of gender differences can facilitate the development of personalized prevention and treatment strategies for different genders, enhancing intervention efficacy [18-21].

Age factor

Age is a key determinant of PSG parameters in snoring patients. Children and adolescents (0-20 years old) generally demonstrate better PSG parameters, likely due to their relatively normal airway structure and robust respiratory regulation function. As individuals age, especially during middle and older adulthood, various bodily functions decline, with the reduction in upper airway regulation being particularly notable. This decline may increase the risk of airway collapse and susceptibility to hypoxia. Furthermore, common age-related phenomena such as muscle relaxation and soft tissue hyperplasia may alter airway structure, further exacerbating snoring. Consequently, age serves not only as an important indicator of snoring risk but also indirectly influences disease severity through multiple mechanisms [22, 23].

Factors influencing AHI severity

AHI is a critical metric for assessing the severity of sleep apnea. Different AHI levels reflect varying stages of the disease and their impact on patient health. As AHI values rise, respiratory function and hypoxia become increasingly impaired, leading to issues such as daytime sleepiness, reduced attention, and heightened cardiovascular disease risk. Research has identified a significant positive correlation between BMI and AHI, underscoring the pivotal role of obesity in sleep apnea pathogenesis [24-27]. Notably, the relationship between age and AHI is complex; while AHI tends to increase with age, substantial individual variation exists. Therefore, age should not be used as the sole criterion for evaluating sleep apnea severity in clinical practice.

Limitations

Although this study provides a relatively comprehensive analysis of PSG parameters in ENT patients with snoring, several limitations warrant acknowledgment. First, the sample source may exhibit geographical bias, as regional differences in physical constitution and lifestyle could influence the generalizability of the findings. For example, dietary habits in certain areas may contribute to higher obesity rates, affecting AHI distribution patterns. Second, the study does not fully account for other factors that may influence PSG parameters, such as medication history and family genetic history. These factors could potentially confound accurate identification of snoring etiology. Future research should expand the sample size and incorporate additional variables for a more thorough and reliable analysis [28].

## Conclusions

By analyzing the PSG parameters of snoring patients in the otorhinolaryngology department across different genders, ages, and AHI levels, we have elucidated the variations and correlations of each parameter among distinct population characteristics. These findings not only enhance our understanding of snoring but also offer critical references for clinicians in early diagnosis, personalized treatment planning, and disease evaluation. In addition, they provide a solid foundation for further investigating the pathogenesis of snoring.
